# Injection, hematoma, abscess, systemic inflammatory response syndrome, stairway to hell: Case report

**DOI:** 10.1097/MD.0000000000038930

**Published:** 2024-07-12

**Authors:** Kemal Gokkus, Baris Sargin, Bahtiyar Haberal, Mehmet Sukru Sahin

**Affiliations:** a Department of Orthopedics and Traumatology, Baskent University, Alanya Research and Practice Hospital, Alanya/Antalya, Turkey; b Baskent University Medical Faculty, Yukari Bahçelievler, Çankaya/Ankara, Turkey.

**Keywords:** case report, dorso-gluteal injection, gluteal abscess, superior glutel artery injury, systemic inflammatory response syndrome

## Abstract

**Rationale:**

Intramuscular injections are routine outpatient procedure performed at healthcare institutions worldwide. In the current literature, there have been very few reports of gluteal superior artery injuries due to incorrect injection techniques. However, no one has ever reported a healthy middle-aged man with systemic inflammatory response syndrome with possible injection-related bleeding from the gluteus superior artery, followed by a hematoma, and then a deep abscess after 3 weeks of not receiving treatment.

**Patient concerns:**

A 40-year-old man presented with pain in his buttock, a fever of 40°, and a lump after a dorso-gluteal injection. (November, 2022) The patient was diagnosed with systemic inflammatory response syndrome due to a deep abscess related to a hematoma caused by a possible superior gluteal artery branch injury.

**Diagnoses:**

He was admitted to our institution with a lump, pain in his buttock, and a fever of 40° after a dorso-gluteal injection. The patient had diffuse swelling and tenderness in the upper-posterior aspect of the gluteal region. Systemic examination revealed yellow sclera and icteric skin appearance. Blood tests showed low hemoglobin levels and increased pre-sepsis parameters (procalcitonin and indirect bilirubin). Pelvic MRI and ultrasonography revealed a gluteal abscess.

**Interventions:**

The patient was transferred to the operating theater, where a curved incision was made behind the trochanter. The gluteus maximus was bluntly dissected, and abscess fluid was drained from the muscle. Continuous bleeding was detected, suggesting iatrogenic superior gluteal artery branch injury at the time of the injection.

**Outcome:**

After drainage and antibiotic treatment, the patient’s parameters normalized within 5 days, and the patient was discharged. The patient’s weekly follow-up examinations were normal, and he was able to walk without a limp. A postoperative visit to the outpatient clinic 2 months after the operation and a telephone call 17 months later showed that the patient was completely healthy and able to work.

**Lessons:**

The dorso-gluteal technique has potential risks, including possible injury to the sciatic nerve and superior gluteal artery and irritation of the subcutaneous adipose tissue. This article aims to highlight the potential risks of a particular technique and advocate the use of the ventrogluteal technique instead of the traditional dorso-gluteal technique.

## 1. Introduction

Intramuscular (IM) injections are routine outpatient procedures performed in healthcare institutions worldwide. The IM route is a useful method for administering medication. It is used when relatively rapid absorption and long-lasting effects are needed. Although the procedure may seem insignificant, it can occasionally lead to significant problems such as abscesses, bacteremia, and widespread sepsis, which can ultimately result in multi-organ failure.^[1,2]^

Several authors have reported the post-injection gluteal abscesses. Nevertheless, no one has ever reported a healthy middle-aged man with pre-sepsis with possible injection-related bleeding from the gluteus superior artery, followed by a hematoma, and then a deep abscess after 3 weeks of not receiving treatment.

## 2. Timeline

2022-11-11 Dorso-gluteal injection at another medical center.

2022-11-13 Pain and tenderness in his left buttock. Several ineffective palliative treatments was prescribed in another center. The accurate diagnosis was overlooked.

2022-11-17 Pain and tenderness in his left buttock. Several ineffective palliative treatments was prescribed in another center. The accurate diagnosis was overlooked.

2022-11-20 The wavy pattern of fever had been initiated that was gradually increase up to 39° to 40°. The empiric antibiotic treatment has been initiated.

2022-11-21 Pelvic X-ray ordered and revealed no pathology. The accurate diagnosis was overlooked.

2022-11-23 Ultrasonography was performed. The abscess has been detected, and the doctors decide to treat with antibiotics.

2022-11-30 Patient admitted to our institution and incisional drainage was performed.

2022-12-05 After drainage and appropriate parenteral antibiotic treatment, the patient’s parameters normalized within 5 days and the patient was discharged.

2023-02-06 A postoperative visit to the outpatient clinic 2 months after the operation, he was completely healthy and able to work.

2024-04-05 After the operation, a telephone call 17 months later showed that he was completely healthy and able to work.

## 3. Narrative

This case report complied with the Declaration of Helsinki, and ethical approval was not required as the study was a case report. Written informed consent was obtained from the patient. This case report was written in accordance with CARE guidelines.

**Figure 1.**
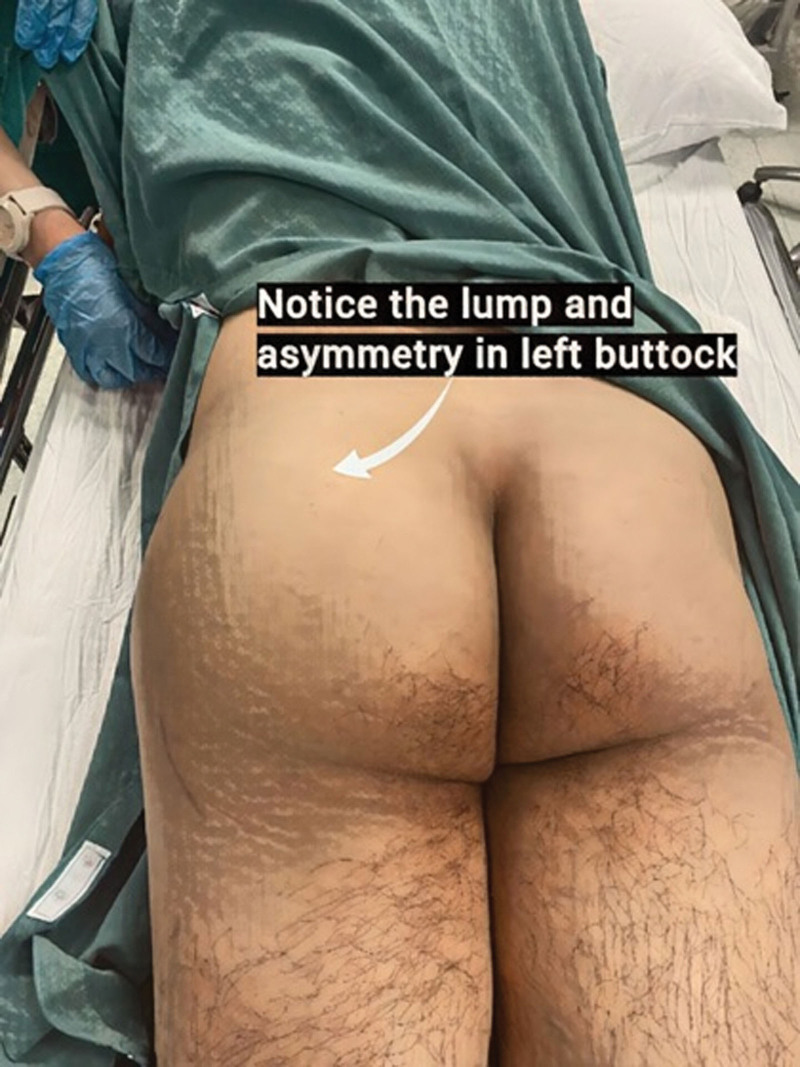
Appearance of the left buttock before the drainage. Notice the lump and asymmetry in the left buttock.

A 40-year-old man presented at our clinic with pain in the left buttock, fever of 40°, and a lump. The pain persisted for 19 days after a dorso-gluteal injection (diclofenac sodium) at another medical center. The pattern of fever pattern was wavy and worsened at night. Upon examination, the patient had diffuse swelling with tenderness in the upper-posterior aspect of the gluteal region. Systemic examination revealed a yellow sclera and an icteric appearance of skin (jaundice) at admission (see Fig. 1).

Routine blood investigations showed Hb% ‐ 7.9 g%, total WBC counts 7.65 cells/mm^3^, differential count-neutrophil 89.45%, lymphocyte 5.42%, monocyte 4.44%, and eosinophil 0.10%, and C-reactive protein was 259.2. It has been observed that the pre-sepsis parameters (procalcitonin: 0.62 ng/mL and direct bilirubin: 3.12 mg/dL, gamma-glutamyl transpherase: 226 U/L) increased. Neutrophil-to-lymphocyte ratio 89.45/5.42. Abdominal ultrasonography revealed no gallstones or biliary tract obstructions. Pelvic MRI and ultrasonography revealed a deep gluteal abscess. The patient was immediately transferred to the operating theater.

**Figure 2.**
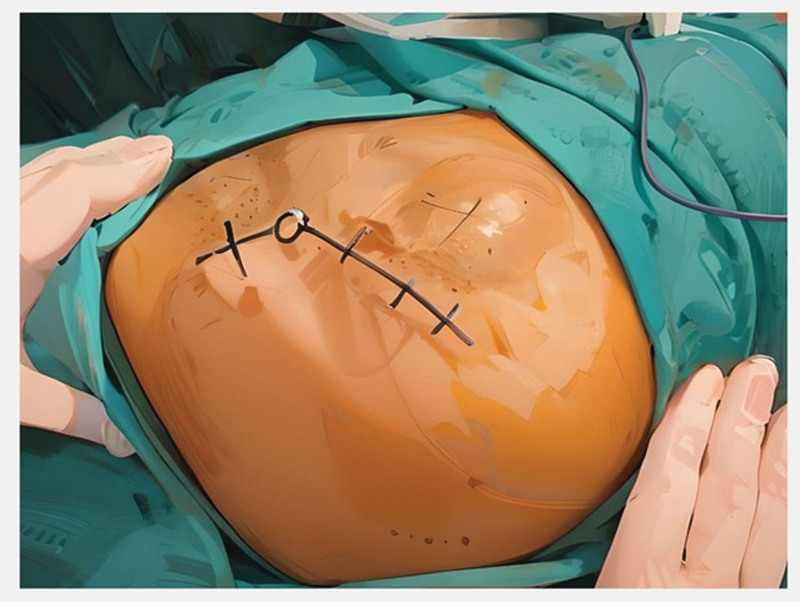
Note the position of the patient (lateral decubitis) and the trochanter-based, posteriorly curved incision.

The posterolateral approach was performed in the lateral decubitus position, and a curved incision was made behind the trochanter through the skin and fascia (see Fig. 2).

**Figure 3.**
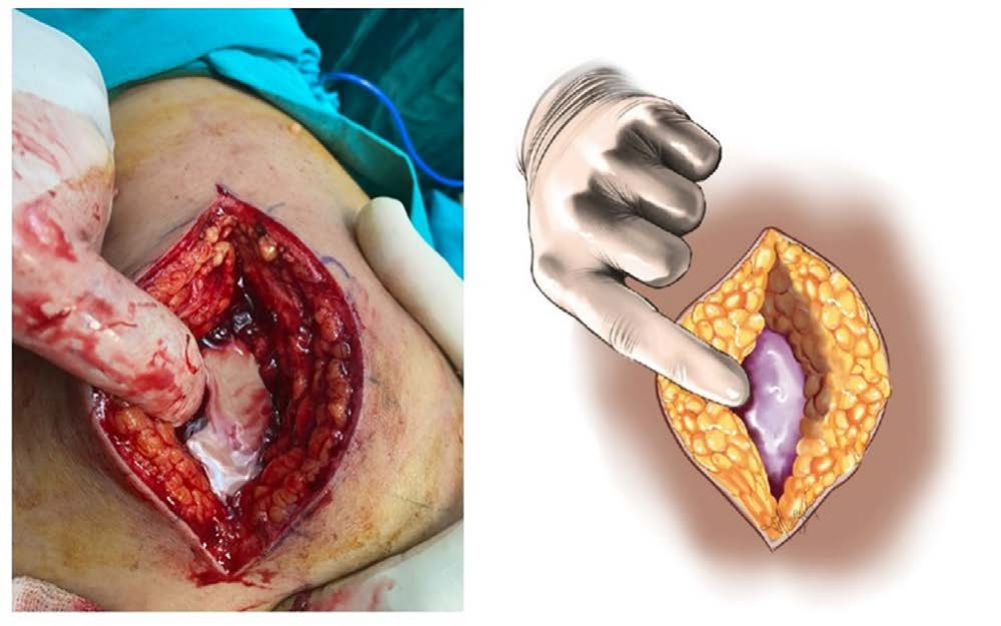
Note the appearance of the abscess under the gluteus maximus. The color of the abscess was whitish yellow color.

The gluteus maximus was bluntly dissected along the fibers. Under the gluteus maximus muscle, abscess fluid (whitish yellow) is drained (see Fig. 3)s.

After drainage, there was still bleeding between the fibers of the gluteus medius muscle, which suggests that the injection caused damage to a branch of the superior gluteal artery. This finding also helps elucidate the exceedingly low hemoglobin levels observed upon admission.

One hemovac drain was left in the pouch, and the layers were sutured anatomically. After a follow-up of 5 days, the drainage through the removal decreased to 5 to 10 mL, and the drain was removed.

The microbiological examination revealed a *Staphylococcus aureus* bacterium.

After drainage and appropriate parenteral antibiotic treatment, the patient’s parameters normalized within 5 days, and the patient was discharged. The patient’s weekly follow-up examinations were standard, and he was able to walk without a limp. A postoperative visit to the outpatient clinic 2 months after the operation and a telephone call 17 months later showed that the patient was completely healthy and able to work.

## 4. Discussion

**Figure 4.**
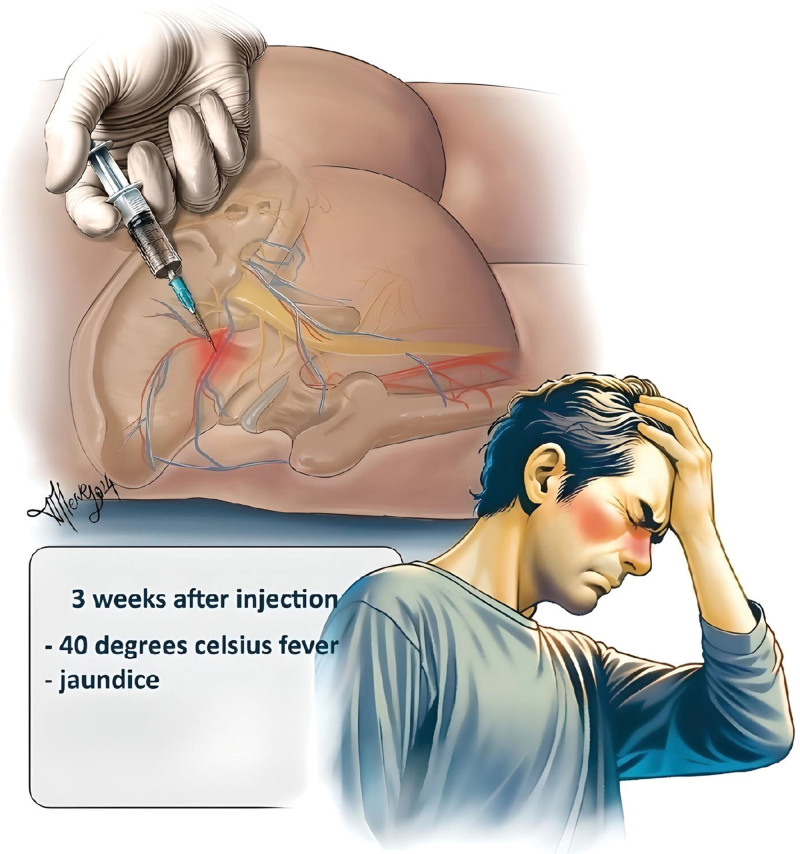
The provided illustration depicts the injury to the superior branch of the gluteal artery resulting from the penetration of the injection tip.

**Figure 5.**
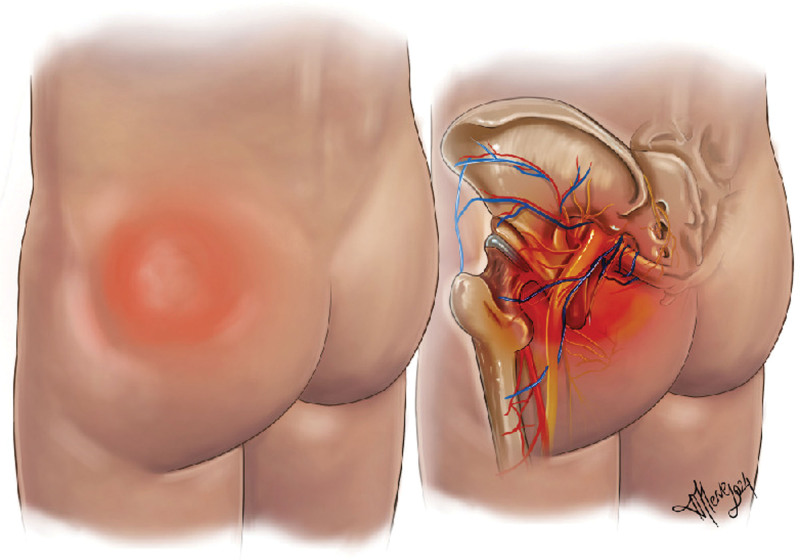
The provided illustration depicts the formation of a hematoma following an injury to a branch of the superior gluteal artery.

The most important finding of this study is that an incorrect injection technique can result in bleeding from the superior gluteal artery, leading to the formation of hematoma. If left untreated, a deep abscess may develop and progress to a pre-septic state. An illustration of the pathophysiology and flow of events is shown in Figures 4 and 5.

In the current literature, there have been very few reports of gluteal superior/inferior artery injury due to incorrect dorso-gluteal injection techniques.^[3–5]^

A literature review revealed a few reports regarding the pre-sepsis/sepsis state after the post-injection abscess.^[1,2]^

Ratzinger et al emphasized the importance of procalcitonin and bilirubin parameters in identifying systemic inflammatory response syndrome in standard-care patients.^[6]^

This is the first case in the current literature to describe both a hematoma due to superior gluteal artery injury and an abscess in a systemic inflammatory response syndrome.

Angela Cocoman and John Murray (2006) preferred ventral-gluteal injections over dorso-gluteal injections. They criticized dorso-gluteal injection because of the potential risk of damaging major nerves and blood vessels, the slower absorption of medication from this site compared to others, and the presence of a thick layer of fatty tissue, which commonly causes issues.^[7]^

In 1983, Muller-Vahl authored a letter to Lancet, highlighting the significance of employing the appropriate technique for IM injections. They suggested that the ventrogluteal method is a safer alternative to traditional injection techniques.^[8]^

Kathleen Greenway (2004) studied using the ventrogluteal site for IM injection. The administration of IM injections is a common nursing intervention; however, the use of the dorso-gluteal site in the United Kingdom is ineffective and potentially dangerous. The ventrogluteal site is a better option, as it provides a consistent layer of adipose tissue and avoids the risk of damaging nerves or blood vessels.^[9]^ Nurses should be taught the ventrogluteal technique along the dorso-gluteal site. The dorso-gluteal site carries risks such as damage to the sciatic nerve, superior gluteal artery, and irritation to the subcutaneous tissue. Teaching the ventrogluteal technique may be challenging, but it is necessary for patient safety.^[9]^ The researchers hypothesized that the usual injector length in the classical upper quadrant gluteal injection approach is insufficient to penetrate the muscle because of the thick fat pad in this area.^[9]^

Mclvor et al reported a case who has been suffered from a gluteal abscess after a triamcinolone injection. Approximately 60 cc of thick green pus was drained, and cultures revealed coagulase-positive *Staphylococcus*.^[10]^

The authors criticized the classical and mostly applied injection method of the upper outer quadrant gluteal method and emphasized its disadvantages.^[10]^

According to the authors, the main mistake in this injection technique is often the administration of the injector somewhat further towards the center. According to their statement, this might potentially harm the gluteal arteries and sciatic nerve.

They cited the Swiss anatomist von Hochstetter, who was credited with ventrolateral injection. The authors also highlighted that the short length of the classical injector was a problem (~3 cm).^[11]^

## 5. Patient perspective

The patient declared satisfaction regarding the pain relief and outcome of the surgery.

## 6. Conclusion

In our country, the dorso-gluteal technique is traditionally taught in medical practice (medical schools) and probably also in nursing schools. In this article, we want to raise awareness of its potential risks and recommend the ventrogluteal technique over the traditional method.

## Acknowledgments

The authors thank the patient for participating and agreeing to publish the report. The article features illustrations created by Merve Evren, a professional medical illustrator. The authors of this article express their gratitude for this remarkable study.

## Author contributions

**Conceptualization:** Kemal Gokkus, Bahtiyar Haberal, Mehmet Sukru Sahin.

**Data curation:** Kemal Gokkus, Baris Sargin.

**Formal analysis:** Kemal Gokkus, Baris Sargin.

**Investigation:** Kemal Gokkus.

**Methodology:** Kemal Gokkus, Mehmet Sukru Sahin.

**Supervision:** Kemal Gokkus, Mehmet Sukru Sahin.

**Validation:** Bahtiyar Haberal.

**Visualization:** Kemal Gokkus.

**Writing – original draft:** Kemal Gokkus.

**Writing – review & editing:** Kemal Gokkus, Mehmet Sukru Sahin.
